# *Fusobacterium* & Co. at the Stem of Cancer: Microbe–Cancer Stem Cell Interactions in Colorectal Carcinogenesis

**DOI:** 10.3390/cancers15092583

**Published:** 2023-04-30

**Authors:** Giovambattista Pani

**Affiliations:** 1 Department of Translational Medicine and Surgery, Section of General Pathology, Faculty of Medicine, Università Cattolica del Sacro Cuore, Largo Francesco Vito, 1, 00168 Rome, Italy; giovambattista.pani@unicatt.it; Tel.: +39-06-30154914; 2 Fondazione Policlinico Universitario A. Gemelli IRCCS, L. go A. Gemelli 8, 00168 Rome, Italy

**Keywords:** colorectal cancer, *Fusobacterium nucleatum*, *Helicobacter Pylori*, intestinal stem cells (ISC), colorectal cancer stem cells (CR-CSC), inflammation, wound healing, Wnt/β-catenin

## Abstract

**Simple Summary:**

Colorectal cancer is one of the most frequently diagnosed and deadly malignancies worldwide, but our understanding of why this life-threatening disease occurs is still limited. With trillions of bacteria inhabiting our intestines, especially the large intestine, where cancer most frequently develops, it is no surprise that gut microbes have been under scrutiny. One of the prime suspect microorganisms is *Fusobacterium nucleatum*, an oral pathogen believed to lodge in colon cancer at its initial stage and foster its progression to full malignancy. Based on a review of the available information, we propose that *Fusobacterium* facilitates colorectal cancer through a misguided attempt to heal the diseased mucosa gone tragically wrong. This provocative view aims at stimulating discussion and putting the healing wound-cancer analogy in the spotlight of future research on the role of gut bacteria in colon malignancy.

**Abstract:**

Adult stem cells lie at the crossroads of tissue repair, inflammation, and malignancy. Intestinal microbiota and microbe–host interactions are pivotal to maintaining gut homeostasis and response to injury, and participate in colorectal carcinogenesis. Yet, limited knowledge is available on whether and how bacteria directly crosstalk with intestinal stem cells (ISC), particularly cancerous stem-like cells (CR-CSC), as engines for colorectal cancer initiation, maintenance, and metastatic dissemination. Among several bacterial species alleged to initiate or promote colorectal cancer (CRC), the pathobiont *Fusobacterium Nucleatum* has recently drawn significant attention for its epidemiologic association and mechanistic linkage with the disease. We will therefore focus on current evidence for an *F. nucleatum*-CRCSC axis in tumor development, highlighting the commonalities and differences between *F. nucleatum*-associated colorectal carcinogenesis and gastric cancer driven by *Helicobacter Pylori*. We will explore the diverse facets of the bacteria–CSC interaction, analyzing the signals and pathways whereby bacteria either confer “stemness” properties to tumor cells or primarily target stem-like elements within the heterogeneous tumor cell populations. We will also discuss the extent to which CR-CSC cells are competent for innate immune responses and participate in establishing a tumor-promoting microenvironment. Finally, by capitalizing on the expanding knowledge of how the microbiota and ISC crosstalk in intestinal homeostasis and response to injury, we will speculate on the possibility that CRC arises as an aberrant repair response promoted by pathogenic bacteria upon direct stimulation of intestinal stem cells.

## 1. Introduction: The Physiology behind Cancer—Embryonic Development and Tissue Repair

Cancer killed about 10 million people worldwide in 2020, with an estimated 19.3 million new cases in the same year [1]. These impressive numbers reflect the complex and multifaceted origin of malignancy, to which genetic and environmental factors contribute through an endless array of molecular interactions and aberrant biological responses, leading to uncontrolled cell proliferation, local invasion, distant dissemination, body wasting, and death [2].

Why is cancer so frequent? Malignancy is broadly recognized as a pathologic distortion of physiological cell programs essential for life and associated with cell multiplication and tissue growth: embryonic development [3], tissue homeostasis, and injury repair [4]. All these programs call upon a unique subset of cells endowed with self-renewal, extended proliferative potential, and multilineage differentiation capacity, indicated as (embryonic or adult) stem cells (SC) [5]. In keeping with cancer being somewhere in between a growing embryo and a repairing tissue, stem or stem-like cells (indicated as cancer stem cells, CSC, or tumor-initiating cells, TIC) have been identified over the last two decades in hemopoietic malignancies and in most solid tumors, including CRC [6]. These “high-rank” tumor cells are believed to account for local tumor recurrence after surgery, chemo-resistance, and metastatic dissemination, thus representing an ideal target for novel therapeutical approaches [7].

Cancer stem cells (CSC) may represent normal resident stem cells undergoing oncogenic transformation or instead arise from the mutation-induced de-differentiation of more mature or even fully differentiated cell elements [8,9]. Indeed, random mutations occurring during the physiologic division of stem cells may account for up to two-thirds of cancer risk variations among tissues. The remainder is related to genetic or environmental factors, such as cigarette smoke, pollutants, radiation, or infectious agents [10,11]. Carcinogens, including viruses and onco-bacteria, may directly target cells endowed with stem cell capacity; alternatively, they could primarily target non-stem tumor cells and turn them into cancer stem-like cells [8].

In this review, we will discuss the role of intestinal stem cells (ISC) and cancer stem cells (CSC) in bacteria-driven colorectal tumorigenesis, focusing on *F. nucleatum* as the pathogen/pathobiont most frequently associated (and mechanistically linked) with the onset and/or progression of this life-threatening malignancy. In doing so, we will elaborate on the analogy between the carcinogenic actions of *F. nucleatum* and the emerging multifaceted interactions between bacteria and intestinal stem cells in the regulation of normal mucosal homeostasis and repair after injury.

## 2. Bacterial Carcinogenesis in Colorectal Cancer

In the early 2000s, the global contribution of biological agents to cancer etiology was estimated at close to 20%, with viruses (12%) taking the largest share and bacteria (6%) following behind. Yet, the only bacteria classified in 2012 as Group 1 (sufficient evidence) carcinogens by the International Agency for Research on Cancer (IARC) were *H. Pylori* for non-cardia gastric carcinoma and low-grade B-cell MALT gastric lymphoma [12]. No biological agent was mentioned in the same publication as causally linked to CRC, except for *Schistosoma japonicum* (classified as group 2A), a worm whose infection predisposes to early-age tumors with a predilection for the distal colon and rectum, likely via chronic bowel injury and inflammation [13].

Although definitive evidence for a bacterial etiology in CRC is still lacking, the idea that intestinal microbes contribute to CRC is far from new [14]. In recent years, the tremendous methodological advances in microbiota profiling, taxonomic and functional characterization, and mechanistic studies on single candidate microbes in model experimental systems have led to exciting new insights into the role of bacteria and their products in CRC [15,16]. These efforts have simultaneously illuminated the exceptional complexity underlying the microbiota–CRC association, which involves both spatial (i.e., tumor versus adjacent mucosa, proximal versus distal colon) and temporal (i.e., precancerous lesions and adenomas versus advanced carcinoma) dimensions. Different yet largely overlapping theoretical frameworks that incorporate single bacterial species and global changes in microbiota composition and diversity (“dysbiosis”) in the classic genetic/epigenetic models of colorectal carcinogenesis have been proposed. The “Alpha-bug” theory posits the existence of a tumor-initiating microbe endowed with genotoxic and pro-inflammatory capacity; the resulting pro-tumoral microenvironment, in turn, shapes cancer microbiota by enriching for pro-oncogenic species at the expense of protective commensals [17]. In the similar “driver–passenger” paradigm, microbiota components found associated with cancer at advanced stages (“passengers”) often differ from those who have initially triggered malignant colonic epithelial cell transformation by means of their oncogenic capacity (drivers) [18]. Notably, (a) driver species may not be detectable in advanced cancers; (b) passenger bacteria are not “innocent” bystanders of tumorigenesis but instead contribute to cancer progression by fostering genomic instability and modulating inflammation and antitumor immunity; and (c) bacterial communities or consortia, besides single bacterial species, may participate in the driver–passenger dynamics [18].

Examples of putative “driver” bacteria include enterotoxigenic *Bacteroides Fragilis* (ETBF), *Enterococcus Faecalis, Salmonella Enteritidis,* and *E.Coli* strains harboring the pks (polyketide kinase) pathogenicity island and producing the genotoxin colibactin [15]. Shared features of these CRC-associated microorganisms are the potential for direct DNA damage, and/or the capacity of hijacking host cell signaling pathways (such as the Wnt/βcatenin-TCF/LEF axis) so as to promote cell proliferation, resistance to apoptosis, and the release of proinflammatory cytokines [16,19]. These characteristics are also shared by *H. Pylori*, the prototype onco-bacterium involved in gastric carcinogenesis [20,21]. It is worth noting that some clearly genotoxic pathogens, such as tilimycin-producing *Klebsiella oxytoca* [22], are tied to acute mucosal damage more than they are to CRC development. Another genotoxic microbe, colibactin-producing *E. Coli,* is weakly carcinogenic per se, but successfully cooperates with proinflammatory ETBF in experimental and likely human colorectal tumorigenesis [23]. Thus, the combination of genomic destabilization, inflammation, and deregulated cell growth, brought about by single microorganisms or microbial consortia, appears crucial for bacteria-driven colorectal cancer.

Unlike drivers, which initiate tumorigenesis within the normal mucosa and may later disappear from the cancer scene, passenger bacteria are highly enriched in tumor tissue compared to the surrounding normal mucosa (or the colon from healthy subjects). In fact, they may have gained a competitive advantage in the newly developed cancerous microenvironment. Remarkable examples are *Streptococcus gallolyticus* (formerly *S. Bovis*), whose clinical detection (i.e., infective endocarditis) has been long considered an alert for undiagnosed colorectal malignancy [24], and *F. nucleatum*, consistently highlighted as a cancer-associated bacteria by high throughput comparative microbiome analysis between CRC and the adjacent non-cancerous tissue [25,26,27].

While probably the most successful colonizer of CRC, *F. nucleatum* is also increasingly recognized as a protagonist in disease promotion and progression (see next paragraph). Therefore, before delving into the central theme of how the crosstalk between bacteria and cancer stem cells contributes to colorectal carcinogenesis, we will review the expanding information on *F. nucleatum* as an intestinal carcinogen, underscoring, where appropriate, similarities and differences with the epitomic cancer-inducing pathogen *H. Pylori.*

## 3. *Fusobacterium nucleatum* and Colorectal Cancer: Inflammation Meets Stemness

### 3.1. F. nucleatum and Human CRC

Evidence in support of a possible causative role for *F. nucleatum* in CRC is burgeoning. This Gram-negative, anaerobic, non-spore-forming bacillus is a major constituent of the dental plaque and has been associated with clinical infections, including periodontitis, obstetric infections, brain abscesses complicating periodontal disease, and bacteremia during prolonged neutropenia [28]. Additionally, *F. nucleatum* is part of a proposed fecal microbial signature for Crohn’s disease [29], a chronic bowel inflammatory disorder that increases the risk for colonic malignancy. Over the last decade, an increased abundance of *F. nucleatum* in CRC tissue compared to the adjacent normal mucosa (or the mucosa of healthy subjects) has been reported in several studies and across patient cohorts belonging to different ethnicities [30]. Moreover, two recent metanalyses confirmed that the detection of *F. nucleatum* in feces or colorectal tissue represents a risk factor for CRC [31,32]. The amount of *F. nucleatum* DNA in CRC tissue is associated with shorter patient survival and may serve as a prognostic biomarker [33,34]. This finding may reflect the enrichment of *F. nucleatum* in CRC of advanced stage [33,35,36], although an increased abundance of *Fusobacteria* has also been reported in the rectal mucosa of adenoma-bearing patients [37], and fecal positivity for *F. nucleatum* and *S. gallolyticus* may be predictive for early-stage CRC [38]. Clinico-pathological correlates of *F. nucleatum* detection in CRC include tumor location (with the proportion of *F. nucleatum*-high CRCs gradually increasing from rectum to cecum [39]), CpG island methylator phenotype (CIMP) positivity, wild type TP53, hMLH1 methylation, positivity for CHD (chromodomain helicase DNA binding protein) 7/8 mutation [40], and microsatellite instability (MSI) [33,40,41]. Moreover, Mima et al. reported an inverse correlation between the amount of *F. nucleatum* and CD3 + T cell density in colorectal carcinoma tissue [42], consistent with the immuno-inhibitory action of this pathogen [43,44]. Along similar lines of evidence, in patients with locally advanced rectal cancer, *F. nucleatum* detection after neoadjuvant chemo-radiotherapy (nCRT) significantly worsened prognosis and increased the risk of relapse, in parallel with a blunted increase of CD8+ T lymphocytes post nCRT [45].

The recently introduced consensus molecular subtype (CMS)-based classification of CRC is increasingly recognized a clinical potential in predicting patient outcomes and response to therapy [46]. Right-sidedness, microsatellite instability, and poor prognosis (especially after relapse) are all features of CMS1 [47]; accordingly, two independent studies have shown an enrichment of *F. nucleatum* in CMS1 compared to other CMSs [48,49], while Ternes et al. found *F. nucleatum* proportion comparably higher in CMS1 and CMS3 (metabolically deregulated) than in CMS2 and CMS4 [50]. Mechanistically, *F. nucleatum’s* capacity to generate genotoxic oxidant species and induce inflammation-associated microsatellite instability [51], coupled with the pathobiont’s ability to promote cancer glutamine metabolism [50], may underlie the above associations with specific CRC CMSs. Alternatively, these associations may reflect a different affinity of *F.nucleatum* for specific tumor microenvironments, marked by distinct mutational, epigenetic, or even microbial [18,52] landscapes.

### 3.2. Mechanistic Studies In Vivo and In Vitro

Studies in rodents have corroborated the idea of *F.nucleatum* acting as a co-carcinogen in colonic tumorigenesis, shedding some light on the possible underlying mechanisms. *F. nucleatum* administration by oral gavage has consistently enhanced intestinal tumor burden in genetically susceptible Apc^Min/+^ mice that bear a constitutive activation of the Wnt/β-catenin oncogenic pathway [53,54]. Interestingly, although *F. nucleatum* infection was associated with a specific cytokine and inflammatory infiltration pattern in tumor lesions, it neither induced colitis nor accelerated tumorigenesis in two distinct mouse models of colitis-associated CRC [53]. This observation suggests that the modality of *F. nucleatum*-driven carcinogenesis may be at least in part different from other CRC-associated bacteria, such as enteroinvasive *E Coli* or *Bacteroides Fragilis*, whose activity appears to be intimately tied with the induction of intestinal inflammation [55,56]. Additionally, although potentially endowed with a genotoxic capacity [51,57], *F. nucleatum* does not appear sufficient to initiate intestinal carcinogenesis in genetically normal mice, unlike chronic infection by *Helicobacter Felis* in C57Bl/6 mice developing gastric cancer [58]. Instead, current evidence points to *F. nucleatum*, at least in animal models, as a cancer promoter [59] that cooperates with genetic and immunological factors to fuel the expansion and progression of an existing malignant lesion, in line with the driver–passenger paradigm (see above) [18].

**Figure 1:**
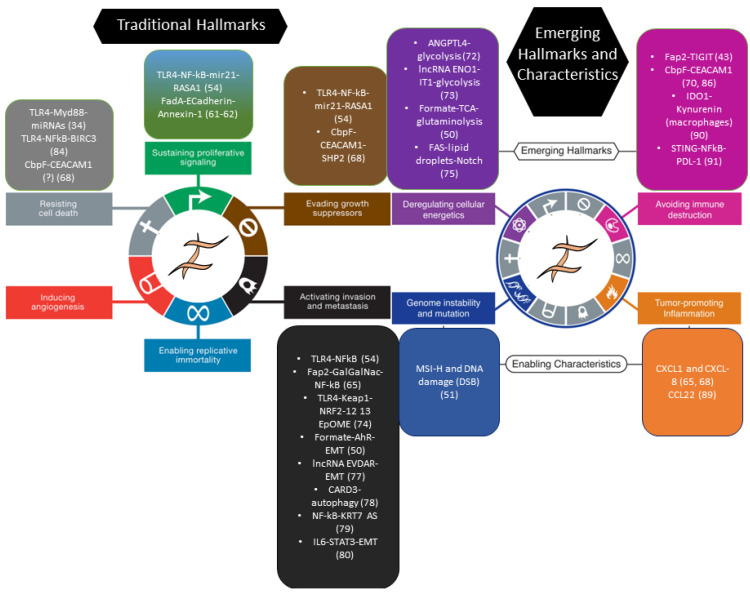
*F. nucleatum* and the hallmarks of cancer in CRC. Figures 1 and 3 from ref. [60] were adapted to outline the documented effect of *F. nucleatum* on CRC cells across the hallmarks and enabling characteristics of cancer, as proposed by Hanahan and Weinberg. Color-matched boxes indicate the hallmark, the molecule/pathway whereby *F. nucleatum* modifies that specific hallmark, and the corresponding reference number in the present article. Note that lists are not exhaustive and reflect the literature selection operated in the main text. DSB: Double strand breaks; EMT: Epithelial-to-mesenchymal transition; 12,13 EpOME: 12,13 epoxyoctadecenoic acid; MSI-H: Microsatellite instability—high. Reprinted/adapted with permission from Ref. [60], 2011, Elsevier.

Along with animal studies, an impressive body of data on the tumorigenic actions of *F. nucleatum* in cultured colorectal cancer cells suggests that this microorganism actively participates in the evolution of the disease. These effects impact nearly all the hallmarks and enabling characteristics of the cancer conceptual framework proposed a few years ago by Hanahan and Weinberg [60] (Figure 1).

*F. nucleatum* has been consistently reported to increase the proliferative capacity of broadly used CRC cell lines, such as HCT116, LoVo, and SW480, both in vitro and upon engraftment in immunocompromised mice [54,61,62]. This effect appears specific for malignant as opposed to non-cancerous adenoma cells, consistent with *F. nucleatum* acting as a “facilitator” rather than an initiator of intestinal carcinogenesis [62]. Mechanistically, Rubinstein et al. found that the fusobacterial adhesin FadA binds to E-cadherin (CDH1) and activates Wnt/β-catenin signaling, causing nuclear translocation of β-catenin and overexpression of inflammatory genes, Wnt genes, and oncogenes c-Myc and Cyclin D1 (CCND1) [61]. Along parallel lines of evidence, Yang and colleagues reported a different circuitry whereby *F. nucleatum* triggers the innate immune Toll-like receptor (TLR) 4-Myd88-NfkB cascade to upregulate *mir21*, which inhibits the expression of the Ras GTPase and growth suppressor RASA1 [54]. Of note, mir21, which is also a target of *H. Pylori* in gastric carcinogenesis [63], facilitates β-catenin nuclear translocation in APC-mutated CRC cells [64]. Thus, *F. nucleatum* appears to target epithelial CRC cells through multiple surface receptors and signaling pathways, that converge on impaired cell cycle control and the activation of an inflammatory response. Consistent with this general theme, Casasanta et al. identified CXCL-1 and CXCL-8 (also known as Interleukin 8) as the principal cytokines/chemokines secreted by epithelial CRC cells in response to *F. nucleatum* binding and infection [65]. These mediators act in an autocrine and paracrine fashion to promote tumor cell migration, while recruiting cancer-associated stromal cells to favor the establishment of a pro-metastatic tumor microenvironment. Data also indicate that chemokine secretion elicited by *F. nucleatum* in cancer cells (but not in tumor macrophages) is mediated mainly by the fusobacterial lectin Fap2 binding to the tumor-specific sugar moiety Gal/GalNac [65]. The same lectin–sugar interaction may account for the enrichment of *F. nucleatum*, an oral pathobiont, in colorectal carcinoma tissue, possibly following systemic hematogenous dissemination [66,67]. In line with these findings, we’ve reported that Gal/GalNac is abundantly expressed on primary CRC-derived spheroids, a population highly enriched in cancer stem-like cells (CR-CSC) [68]. Fap2/Gal-GalNac binding could therefore mediate a direct interaction between *F. nucleatum* and undifferentiated cancer precursors. In the same CSC population, *F. nucleatum* also engages the carcinoembryonic antigen family cell adhesion molecule (CEACAM)-1, leading to the dissociation of the CEA-associated protein tyrosine phosphatase SHP-2. In turn, relief from phosphatase control unleashes a growth factor-like tyrosine phosphorylation cascade that eventually activates ERKs [68]. *F. nucleatum* binding to CEACAM-1 occurs through the trimeric autotransporter adhesin CbpF1 [69], contributing to this microorganism’s T-cell inhibitory action [70]; accordingly, CbpF is sufficient, when recombinantly expressed in *E. Coli*., to activate the CEACAM-1-phoshotyrosine-ERK axis in CRC spheroids. We also confirmed, in agreement with previous reports on non-stem CRC cell lines [54,61,62,65], that CSC exposure to *F. nucleatum* activates Wnt/β-catenin-TCF/LEF signaling, NF-kB transcriptional activity, and a cytokine response selectively involving CXCL-1 and CXCL-8. Although not definitively proven, these oncogenic and proinflammatory responses of CR-CSCs to *F. nucleatum* are likely to be mediated, at least in part, by the CbpF-CEACAM-SHP2 axis [71].

Metabolic rewiring and facilitation of invasiveness/metastasis are additional relevant aspects of *F. nucleatum*’s pro-tumorigenic action on CRC cells. Zhang et al. identified angiopoietin-like factor 4 (ANGPTL4) as the molecule responsible for the glycolytic switch in *F. nucleatum*-infected DLD-1 cells. The authors demonstrate that enhanced glycolysis is instrumental to the intracellular persistence of *F. nucleatum* in an aerobic environment, suggesting that metabolic derangement and the ensuing enhanced proliferative capacity may represent collateral cellular consequences of a bacterial survival strategy [72]. Along with these observations, Hong et al. found exposure to *F. nucleatum* to be associated with increased glycolytic metabolism in DLD-1 and HCT116 cells, based on lactate production and upregulation of key glycolytic enzymes, including enolase (ENO-1) [73]. Adding to the relevance of this finding, the authors also found a correlation between high tumor glucose metabolism, assessed by ^18^F-FDG PET/TC, and fusobacterial load in tumor tissue from 33 CRC patients. The study also highlights a novel epigenetic circuitry for the *F. nucleatum*-induced glycolytic switch, which involves a previously unknown lncRNA (ENO1-IT1) and the associated histone acetyltransferase KAT7 [73]. Again, on metabolic rearrangements elicited by *F. nucleatum* in CRC cells, Kong and colleagues reported that *F. nucleatum* triggers a TLR4-Keap1/NRF-2 cascade to induce the expression of the cytochrome p450 monooxygenase and increase the synthesis of epoxyoctadecenoic acid (12, 13 EpOME) from polyunsaturated fatty acids. 12-13 EpOME promotes cancer cell migration and epithelial-to-mesenchymal transition (EMT) in vitro and enhances metastatic cell capacity in vivo [74], thus linking *F. nucleatum*-driven altered cell metabolism with malignancy. The interplay between *F. nucleatum* infection, tumor metabolism, and malignant phenotype is also central to an elegant study by Elizabeth Letellier and colleagues, who used a sophisticated bacteria-CRC cell co-culture platform to investigate *F. nucleatum*-CRC co-metabolomics. Data revealed a key role for bacteria-derived formate as a carbon source that feeds into the cancer cells’ tricarboxylic acid (TCA) cycle and drives glutamine metabolism at the expense of glycolysis. In parallel, formate activates the aryl hydrocarbon receptor (AhR) signaling pathway, so as to promote cell migration and elicit cancer stem cell traits, including high metastatic capacity and active Wnt signaling [50]. By focusing instead on lipid metabolisms, Liu et al. reported that *F. nucleatum* enhances colorectal CSC self-renewal, organoid formation, and tumorigenic capacity in vivo by promoting CPT1 (carnitine palmitoyl transferase)-1 expression and fatty acid oxidation (FAO) [75] in mitochondria. Surprisingly, the opposite metabolic response (i.e., fatty acid synthesis) incited by *F. nucleatum* allowed non-stem cancer cells to gain CSC features through the lipid droplet-dependent degradation of the Notch pathway inhibitor Numb [75].

Thus, while different co-culture conditions may justify the divergent metabolic changes (i.e., glutamine or fatty acid mitochondrial metabolism versus glycolysis) elicited by *F. nucleatum* in CRC cells, the above studies collectively highlight a profound link between metabolic reprogramming and tumor cell acquisition of migratory/invasive capacity, a mesenchymal phenotype, and overall traits reminiscent of cancer stem cells [7].

Additional molecular studies, echoing clinicopathological evidence from human CRC [76], have specifically focused on the link between *F. nucleatum* infection and epithelial-to-mesenchymal transition (EMT), cell invasiveness, and metastasis. Proposed mechanisms for *F. nucleatum*-triggered EMT and metastatic behavior include the upregulation of the long non-coding (lnc) RNA EVADR (which stabilizes the EMT-related transcription factors Snail, Slug, and Zeb 1/2) [77] and the induction of autophagy through the bacterial sensor and immune/inflammatory kinase CARD3/RIP2 [78]. Along similar lines, Chen et al. used RNA sequencing to identify lncRNA Keratin7-antisense (KRT7-AS) as an *F. nucleatum*-induced gene in CRC cells; KRT-7 AS up-regulation by *F. nucleatum* is mediated by the proinflammatory factor NF-kB, and is essential for infection-induced cancer cell migration in vitro and metastasis in vivo [79]. Likewise, SW480 and HCT-116 CRC cells exposed to F. *nucleatum* were found to undergo EMT and acquire cancer stem cell characteristics (including growth in spheroids) through an “inflammatory” IL-6/STAT3 autocrine loop [80].

**Figure 2:**
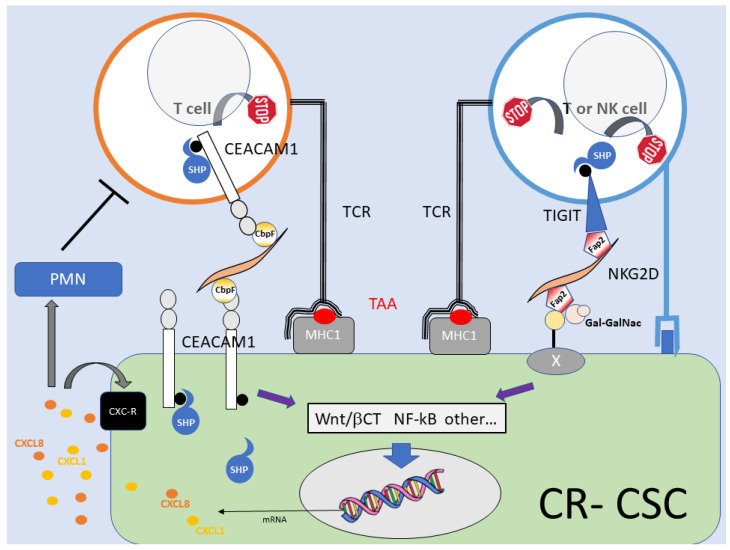
Coordinated pro-tumorigenic and immuno-inhibitory activities of CSC-bound *F. nucleatum* via the CbpF and Fap2 adhesins. A model for simultaneous CR-CSC activation and cancer immune evasion by *F. nucleatum*. Left: *F. nucleatum* engages CEACAM-1 in CSCs (dissociation of the SHP 1/2 phosphatases leads to cell activation) and in TAA-specific T lymphocytes (recruitment of the SHP 1/2 phosphatases downregulates TCR signaling). Right: Fap-2 triggers CSCs via Gal-GalNac glycoproteins while blocking T and NK cells through TIGIT and its effector SHPs, which target the TCR and the activating NK receptor (e.g., NKG2D). CSC activation culminates in the secretion of chemokines that promote CSC motility/invasion and recruit (immunosuppressive) neutrophils into the tumor microenvironment. See text for details and references. CbpF: CEACAM-binding protein fusobacterial; Fap-2: Fibroblast activation protein—2; MHC-1: Major Histocompatibility Complex, Class I; NKG2D: Natural killer receptor G2D; PMN: polymorphonuclear cells; SHP: Src-homology 2 domain (SH2)-containing protein tyrosine phosphatases (SHP-1 and SHP-2); TAA: Tumor-associated antigen; TCR: T cell receptor. The black circle on CEACAM1 and TIGIT indicates the phosphorylated ITIM.

Just like EMT and metastatic dissemination, chemoresistance and tumor cell capacity to evade the immune system are clinically relevant aspects of CRC progression [81], as well as typical traits of cancer stemness [7,82,83]. *F. nucleatum* abundance correlates with the risk of tumor recurrence after chemotherapy [34], and *F. nucleatum* infection reduces the sensitivity of colorectal cancer cell lines to standard CRC chemotherapeutics in vitro. In particular, induction of autophagy (and the consequent prevention of drug-induced apoptosis) via reduced expression of miRNA-4802 and 18a* [34], and upregulation of the apoptosis inhibitor protein BIRC3 through NF-kB [84] have been reported as distinct and potentially independent effector mechanisms for resistance to cell death downstream of TLR4 engagement. Along similar lines, increased resistance of *F. nucleatum*-treated primary spheroid cultures of colorectal CSC to oxaliplatin correlated with enhanced Wnt/β-catenin activity [68]. Interestingly, *F. nucleatum* reportedly promotes cell death instead of survival in normal (i.e., non-cancerous) intestinal epithelial cells, thus favoring chronic inflammation in ulcerative colitis [85]. On the immunological side, the immunosuppressive action of *F. nucleatum* within the CRC microenvironment [42,45] has been addressed in detail. *F. nucleatum* directly engages two distinct inhibitory receptors on NK cells and T lymphocytes (TIGIT, [T cell immunoreceptor with Ig and ITIM domains] via the adhesin Fap-2 [43], and CEACAM-1 via CbpF [69,70]) to downregulate adaptive immune responses [86], suggesting that *F. nucleatum*-coated tumors may be facilitated in immune evasion. Likewise, colorectal CSC, which are intrinsically immune-resistant [87], may be further shielded from immune attack by binding *F. nucleatum* [68]. Intriguingly, the same CbpF-CEACAM-1 signaling axis that inhibits T cell responses also activates CSCs [68]. (Figure 2)**.** In addition, *F. nucleatum*-exposed CRC cells, including CSCs, release chemokines (CXCL1, CXCL 8, CCl22) capable of recruiting neutrophils and favor the establishment of a tumor-suppressive environment [88,89]. As part of the same immunoevasion strategy, *F. nucleatum* induces the enzyme indoleamine 2,3-deoxygenase (IDO-1) in infected macrophages, depleting tryptophane in the tumor microenvironment in favor of the T cell-inhibitory metabolite Kynurenin [90]. Additionally, of note, *F. nucleatum* upregulates PDL-1 via STING and NF-kB in CRC cells, thus indirectly triggering the PDL1-PD1 immune checkpoint. The silver lining is that, by doing so, *F. nucleatum* may also enhance the efficacy of PD-L1 blockade in immunotherapy [91]. Collectively, these data indicate that multiple immunomodulatory activities contribute to CRC promotion by *F. nucleatum*, in a fashion possibly circumventable by immune checkpoint blockade [89].

In summary, mechanistic studies have revealed a remarkable capacity of *F. nucleatum* to remodel the CRC cell phenotype towards increased malignancy and stem-like features; this occurs via a diverse array of ligands, receptors, and molecular circuitries at the intersection of developmental (Wnt/β-catenin, Notch) and inflammatory (TLR/NLR, CARD3, NF-kB, NRF2) signaling networks. Intriguingly, the same two networks orchestrate intestinal mucosa repair in response to injury [92,93,94].

## 4. How Do Bacteria Talk to Intestinal Stem Cells (ISC)?

Intestinal stem cells (ISC) support the extraordinary self-renewal capacity of gut mucosa through robust proliferation and differentiation into a variety of daughter lineages, including absorptive enterocytes, mucus-producing goblet cells, entero-endocrine cells, and Paneth cells. Unlike the other three cell types that form from trans-amplifying cells at the crypt–villus junction, Paneth cells reside at the bottom of the crypt, intermingled with ISCs, to which they provide mechanical and trophic (“niche”) support. However, Paneth cells are missing in colon crypts, where only two differentiated cell types, enterocytes and goblet cells, are found. ISCs have been identified with the actively cycling Lgr5+ crypt base columnar (CBC) cells; another cell type, mitotically quiescent and marked by BmI1 expression, located just above the crypt base (position +4) may also serve as an alternative ISC pool in case of mucosal injury and replenish lost Lgr+ cells [95].

Elegant studies in *Drosophila* have provided invaluable information on how resident microbial communities and infectious pathogens modulate the regenerative activity of intestinal stem cells (ISC) [96]. In the adult *Drosophila* midgut, ISC activity is maintained by the coordinated action of multiple signaling pathways (JAK-STAT, Wingless/Wnt, and EGFR), triggered by an array of soluble factors released by the surrounding visceral muscle and mature enterocytes [97,98]. Ingestion of non-lethal pathogens (such as *Erwinia carotovora carotovora, Ecc15*) results in a robust proliferation of crypt stem cells, aimed at repairing mucosal damage and restoring intestinal barrier integrity. In this setting, ISC proliferation is triggered by the cytokine-like paracrine factor Upd3 (Unpaired 3), released by damaged enterocytes, and is mediated by the JAK-STAT pathway; in parallel, the Janus kinase (JNK) is also activated by the Duox-dependent generation of reactive oxygen species (ROS), and contributes, together with EGF receptor (EGFR) activation by visceral muscle-derived EGF-like ligands, to ISC proliferation and differentiation in response to infection [99,100]. Epithelial barrier reconstitution, so orchestrated by ISCs, is necessary for the proper recovery of flies after infection with Ecc15 [99]. The “normal” ISC renewal activity also requires baseline bacterial stimulation by the indigenous microbiota, and is reduced in *axenic* (germ-free) flies. Conversely, microbial overgrowth in immunodeficient mutant *Drosophila* leads to ISC hyperproliferation, aberrant differentiation, and a dysplastic gut epithelium [99]. These changes resemble age-dependent changes of intestinal architecture in flies [101], and preneoplastic lesions in mammals [94]. ISC hyperproliferation in the above setting requires the Wingless/Wnt pathway [98], and is counteracted by Delta/Notch differentiative cues; remarkably, inactivation of the latter control circuit, compounded by stress signals (Upds) from enteric bacterial infection, is sufficient to drive ISC-derived intestinal tumors without the need for additional mutations in growth signaling cascades [102].

**Figure 3:**
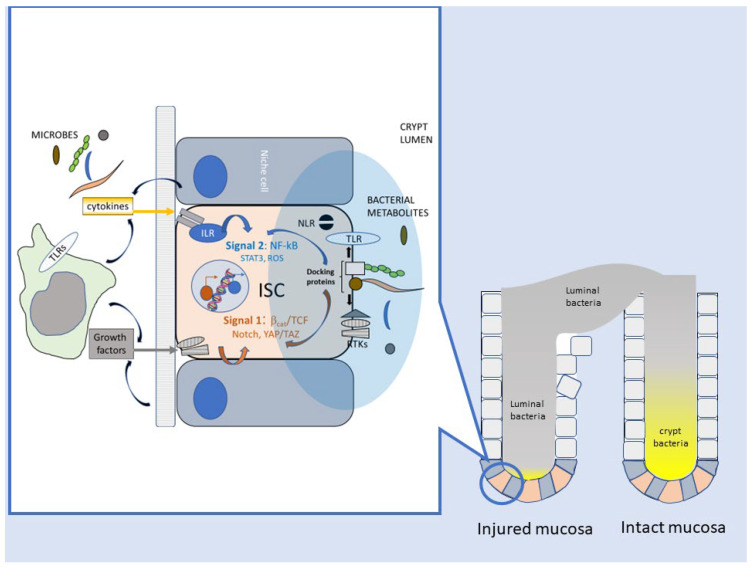
Direct and indirect mechanisms for ISC activation by bacteria. Multimodal interaction of bacteria and intestinal stem cells in mucosal homeostasis and repair. Model built on information from *Drosophila* and the mammalian gut. Mucosal damage changes the microbial ecology of the crypt and possibly allows for bacterial translocation to the stroma. Direct interaction of bacteria and bacterial wall components (i.e., peptidoglycan, muramyl dipeptide, lipopolysaccharide) with pattern recognition receptors (TLRs, NLRs) or specific docking proteins/sugars (i.e., CEACAM-1, E-cadherin or Gal-GalNac) on ISCs activates downstream signaling along the two main axes of RTK-β catenin (signal 1, proliferation/expansion) and NFkB/STAT (signal 2, survival, inflammation). Indirect effects involve the release of growth factors and cytokines by bacteria-stimulated inflammatory/immune cells or niche cells. Additionally, ISC responses can be modulated by microbial metabolites (and their changes due to subversion of the crypt microbiota) acting on ISCs or the surrounding cells. Signaling pathways are indicated schematically. Signals 1 and 2 underscore the analogy between ISC activation and lymphocyte dual signaling (antigen-specific proliferation + inflammatory co-stimulation) during the adaptive immune response (see main text). NLR: Nod [nucleotide binding oligomerization domain]-like receptors; RTK: Receptor tyrosine kinase receptors.

Thus, several lines of evidence link, in the *Drosophila* model, bacteria–host interactions and their imbalances with perturbed ISC homeostasis, enhanced proliferation, and the potential for malignant transformation. These biological responses are mediated by innate immune and developmental signaling cascades, responsive to factors originating primarily from damaged enterocytes or the niche microenvironment. However, ISCs and their daughter cells can also directly sense bacterial components (i.e., peptidoglycan) via G-coupled membrane and cytosolic receptors [103] (Figure 3)**.**

In mammals, commensal microorganisms contribute to the completion of intestinal development after weaning, as suggested by initial observations of overall reduced intestinal cellularity and absorptive surface area in germ-free (GM) mice [104]. More recently, seminal studies on mice deficient in Toll-like receptors 2 or 4 and in Myd88, a nodal adapter downstream of TLR-dependent bacterial signaling [105], have revealed the importance of this cascade in intestinal mucosa homeostasis and repair capacity after chemical or radiation damage [106]. Of note, the TLR-Myd88 signaling axis also promotes spontaneous carcinogenesis in the APCmin mouse models, mainly by impinging on the expression of progression-related modifying genes such as Cox-2 [107]. While these intriguing observations do not allow us to conclusively determine whether epithelial growth in both reparative and tumor settings was directly regulated by bacterial cues or indirectly modulated through stromal/inflammatory cells and their secreted factor, a cell-autonomous connection between TLR-NFkB signaling and intestinal epithelial cell (IEC) proliferation and survival has been, in the meantime, convincingly demonstrated [108,109]. Moreover, villin-driven transgenic overexpression of TLR4 in the mouse intestine led to IEC hyperproliferation, duodenal crypt elongation, and expansion of a Lgr+, stem-like cell population [110]. Additionally, the chemical carcinogen AOM was sufficient to drive colon carcinogenesis in villin-TLR but not WT mice. Interestingly, these changes coincided with increased activity of the Wnt/β-catenin cascade, a pathway pivotal to the maintenance of normal and cancer ISCs [111,112]. In further support of the relevance of this finding, mouse Lgr+ intestinal stem cells have been reported to express TLR4 [113].

A recently published study by van der Post et al. has specifically addressed the mechanistic link between microbiota, TLR signaling, and the proliferation of ISC [114]. The authors found that the ROS-generating membrane oxidase NOX-1 is expressed in mouse colonic epithelium selectively in cycling Lgr5+ stem cells, where it potentiates mitogenic signaling downstream of EGFR via an H_2_O_2_-mediated redox switch. Importantly, NOX-1 is induced in quiescent cells by LPS through the TLR4-Myd88-NF-kB cascade, thus adapting the ISC proliferative response to bacterial density in the intestinal crypt. The generation of ROS by Rac-1, a component of NOX family oxidases [115], is also necessary for the expansion of LGR5+ cells following the deletion of the tumor suppressor APC (and consequent hyperactivation of the *Wnt* pathway) [116], suggesting that bacterial stimuli synergize, through the TLR-ROS-EGFR axis, with genetic or epigenetic changes occurring in stem cells during colorectal tumorigenesis.

Along similar lines of investigation, Nigro et al. analyzed the cytoprotective activity exerted by bacterial peptidoglycan (PGN) on ISC [117]. The breakdown of PGN, a major component of the bacterial wall, generates natural agonists for intracellular pattern recognition receptors belonging to the nucleotide-binding oligomerization domain (NOD)-containing family [105]. NOD2, in particular, is abundantly expressed in Lgr5+ ISC, and application of the bacterial ligand muramyl-dipeptide (MDP) enhances the yield of organoid formation in vitro and accelerates mucosal healing in vivo after oxidative damage by doxorubicin, indicating increased stem cell survival after an otherwise lethal oxidative insult [117]. Mechanistically, MDP triggers NOD2 and the autophagy protein ATG16L1 to initiate the autophagic removal of damaged mitochondria and decrease mitochondrial ROSs responsible for ISC apoptotic death [118]. Interestingly, this effect occurred independent of changes in cell proliferative capacity and was specific for MDP-NOD2, nor was it elicited by other bacterial ligands (such as lipoteichoic acid, LPS, or flagellin) recognized by TLRs. NOD2 and ATG16L1 genetic variants are epidemiologically linked to inflammatory bowel disease [119], which increases the risk of CRC [81]. Moreover, NOD2 suppresses colorectal tumorigenesis by downregulating the TLR pathways [120]. It remains, however, to be established whether these associations between innate immunity and cancer reflect deranged bacterial signaling in ISC as opposed to other intestinal crypt cells [121]. It is also of note that bacterial recognition by intestinal stem cells can elicit opposite changes and downstream consequences of intracellular ROS: an increase (via TLR-NOX1) for ISC expansion [114] or a decrease (via NOD2 and mitophagy) for stem cell cytoprotection [118]. This is in agreement with the well-established notion that intracellular oxygen species trigger divergent cell responses based on their amount, kinetics, and site of generation [122].

While the above examples clearly show that innate immune pathways for bacterial sensing are active in mammalian ISC and modulate mucosal homeostasis and repair, the selectivity of these responses for specific pathogenic and/or commensal bacterial species remains an open issue. However, although PRR receptors recognize highly conserved molecular structures (like those on LPS or PGN) shared by most bacteria, their subcellular distribution (i.e., apical versus basolateral or intracellular versus surface-exposed) may help cells discriminate against invasive microorganisms [123]. Moreover, the crypt microbiota, to which ISCs are potentially exposed, is qualitatively different, especially in the colon, from the luminal flora [124,125] and, interestingly, resembles the restricted microbiota found in the midgut of invertebrates. A modification of such “crypt-specific core microbiota” (CSCM) could timely signal to stem cells the loss of crypt physical integrity or a disruption of its physiological oxygen zonation [126], so as to trigger the regenerative switch. Hopefully, future research will shed light on these still-speculative scenarios (Figure 3).

Metabolomic studies have revealed an additional level of bacteria-stem cell communication besides the activation of “canonical” innate immune signaling. Through an unbiased screening of microbiota-derived metabolites, Kaiko et al. identified the fiber fermentation product butyrate as a potent inhibitor of intestinal stem/progenitor cell proliferation and regenerative capacity [127]. Butyrate acts as a histone deacetylase (HDAC) inhibitor on crypt stem cells, leading to an increased recruitment of the transcription factor Foxo3a to the promoter region of negative cell cycle regulators. Since mature colonocytes, unlike ISCs, oxidize butyrate in the Krebs TCA cycle as an energy source, the authors propose an elegant model whereby mature enterocytes actively degrade bacteria-derived butyrate at the top of the crypt so as to prevent its diffusion to the bottom, where ISCs reside, thus creating a metabolic barrier that preserves ISC activity. Conversely, mucosal damage allows stem and progenitor cell exposure to butyrate, which delays epithelial regeneration [127]. Although this finding is at odds with the need for regenerative activity following mucosal insult, a similar ISC “paralysis” in response to overwhelming epithelial injury by lethal pathogens also occurs in *Drosophila* [96], and the detection of cytosolic double-strand DNA by inflammasome component AIM2 decreases ISC proliferation in mouse intestine to possibly avoid the expansion of genetically damaged or infected cells [128]. Interestingly, ISC blockade by butyrate may fail in genetically initiated cells [129], thus contributing to intestinal carcinogenesis in the context of chronic mucosal damage.

Unlike butyrate, lactate, produced by symbionts such as *Bifidobacteria* and *Lactobacilli*, promotes ISC expansion and protects mice from gut damage inflicted by radio-chemotherapy [130]. This occurs by direct lactate sensing (via the G-coupled receptor Gpr81) in Paneth and stromal cells, which release *Wnt* factors to activate ISCs. However, crypt stem cells are also a direct target of lactate: Rodiguez-Colman and Burgering have shown that lactate produced by Paneth cells through glycolysis fuels oxidative phosphorylation (OXPHOS) in neighboring ISCs, favoring their differentiation into mature crypt cells via a redox signal involving mitochondrial ROS and the JNK kinase [131]. Thus, these two sets of observations converge on lactate as a critical regulator of ISC activity, acting via direct and indirect routes to balance ISC proliferation and renewal with the need for downstream differentiation and maintenance of mucosal integrity.

The delicate equilibrium between self-renewal and differentiation of ISC, crucial to crypt homeostasis, is also modulated by microbial and dietary compounds acting through the aryl hydrocarbon receptor (Ahr) pathway [132]. Mice lacking AhR in the intestinal epithelium display impaired gut barrier integrity upon infection by the pathogen *Cytrobacter Rodentium*, allowing bacteria to disseminate to the liver and spleen and accelerate mortality. Mechanistically, unrestrained *Wnt* activity in the absence of AhR promotes ISC hyper-proliferation, impaired differentiation, and a dramatic failure in mucosal repair. Moreover, independent of infection, AhR-deficient aged mice show impaired intestinal differentiation and subclinical inflammation and are exceptionally prone to experimentally induced carcinogenesis [132]. In keeping with these findings, the gut microbiota’s defect in metabolizing tryptophan into Ahr ligands has been linked to intestinal inflammation in mice and humans [133,134]. Although Ahr signaling in immune cells is arguably important in these disorders [135], impaired ISC responses to leukocyte-derived factors and delayed mucosal healing may also play a role [136]. It should also be noted that microbial sensing through the AhR may have opposite effects in malignant compared to normal intestinal stem cells: in fact, AhR stimulation by formate-producing *F. nucleatum* activates Wnt and increases CSC-like features in colorectal cancer cells [50], and a similar pro-malignant effect of AhR ligands has been reported in the breast cancer cell line MCF-7 [137]. This apparent contradiction deserves to be further clarified, as AhR ligands have been proposed as potential anticancer agents [138].

Besides the direct influence of resident bacteria and their products on intestinal stem cells, inflammatory mediators released by other epithelial and immune components of the crypt microenvironment also shape the mucosal adaptive response to microbes. Interleukin 22 released by innate lymphoid cells (ILC) 3 directly stimulates ISC proliferation via phosphorylation of STAT-3, independent of the Paneth cell niche [136]. Similarly, upon experimental damage, interleukin 6 is upregulated in the mouse intestine and promotes intestinal regeneration and aberrant epithelial differentiation by the parallel activation of inflammatory (STAT3) and developmental (Yes-activated Protein [YAP] and Notch) effectors downstream of the cytokine transducer gp130 [139]. The role of inflammatory cytokines in connecting mucosal damage to ISC activity in the mammalian gut is evolutionarily conserved and recapitulates, at a higher level of tissue complexity, the enterocyte-to-ISC signaling axis mediated by Upd factors during *Dropsophila* midgut regeneration (see above) [96]. For a comprehensive overview of the role of “reparative inflammation” in intestinal regeneration, the reader is referred to the aforementioned review by Karin and Clevers [92].

It is finally possible that bacteria impact ISC activity by altering the physiological cell-to-cell communication within the stem cell niche. In calorie-restricted mice, Paneth cells increase ISC proliferation by the paracrine release of bone stromal antigen 1 (Bst-1), an ectoenzyme that produces the paracrine factor cyclic ADP ribose (cADPR) [140]. cADPR, in turn, activates stem cells through the Sirtuin 1 (SIRT 1)-mediated deacetylation of the mTOR substrate S6-kinase, which favors mucosal growth in response to nutrient shortage [141]. Some bacteria produce cADPR through TIR domain-containing proteins as part of their antiviral response [142], and evidence exists for these bacterial metabolites being altered in malnutrition-associated dysbiosis [143]. Thus, although experimental proof is still missing, it is tempting to speculate that protumoral changes in microbiota composition may impact mucosal homeostasis and possibly promote unchecked ISC proliferation via cADPR-dependent interference with Paneth–ISC communication.

## 5. Bacteria and CRC: Intestinal Repair Gone Awry?

The evidence discussed above clearly identifies the microbiota as a critical regulator of mucosal homeostasis and repair in the mammalian intestine and model organisms. Given the relatively low accessibility of the crypt base to the luminal content, bacteria detection by ISCs relays an early signal of mucosal damage and barrier breach, triggering the repair program as an innate defense strategy aimed at restoring gut barrier integrity. ISC activation occurs through an intricate and evolutionarily conserved signaling network comprising the Wnt/β-catenin cascade, immune receptor signaling upstream of the transcriptional regulators NF-kB and STAT3, and transduction pathways triggered by oxidative (ROS-JNK, ROS-NRF2) and mechanical (Hippo-YAP/Taz) stress (see above) (Figure 3). Extensive or protracted injuries that overwhelm ISCs capacity can also activate alternative regenerative strategies under a similar combination of growth and inflammatory stimuli; these include the dedifferentiation of mature enterocytes [128] and the recruitment of bone marrow-derived pluripotent precursors [58]. Additionally, irrespective of whether bacteria are causes (pathogens) or simply reporters (misplaced commensal microbiota) of mucosal damage, they can also contribute to the resolution of inflammation by promoting “type 2” immunity (anti-inflammatory and reparative) and activating immune checkpoints [144]. Although this can be viewed as a bacterial strategy to evade immunity, mucosal healing benefits from the limitation of antibacterial responses and the accompanying collateral damage to host tissues [145].

**Figure 4:**
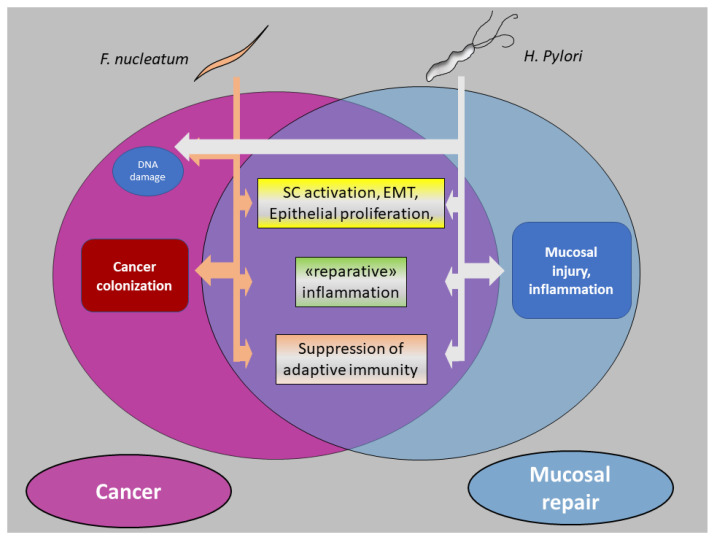
Bacterial carcinogenesis: *F. nucleatum* versus *HP***.** Epithelial proliferation (triggered by stem cell recruitment and «stem-like» conversion [EMT] of mature enterocytes), together with «reparative» inflammation and an immunosuppressive microenvironment, lies at the intersection of intestinal mucosal repair and GI cancer development. *F. nucleatum* and *H Pylori* both activate this prototypical tissue response module, although with different modalities and in distinct phases of carcinogenesis. *HP* displays a higher destructive potential in normal mucosa and triggers the inflammation–atrophy–metaplasia–cancer sequence, fueled by DNA damage and genomic instability. Although potentially harmful for normal mucosa, *F. nucleatum* does not initiate colorectal carcinogenesis but is preferentially recruited to the regenerating/malignant microenvironment, possibly via oxygen-related tissue changes (see text); *F. nucleatum*-induced genomic instability (MSI) may still contribute to cancer evolution. Both models of bacterial carcinogenesis hinge on stem/stem-like cells as emerging players at the forefront of the host–pathogen interface. EMT: Epithelial-to-mesenchymal transition.

We believe that the mechanisms underlying bacterial carcinogenesis, described in the first part of this article, largely overlap those involved in the microbial regulation of mucosal repair along the three axes of Section 5.1, enhanced stemness of epithelial cells; Section 5.2, reparative inflammatory responses; and Section 5.3, downregulation of adaptive immune reactions (Figure 4). Along this line of thinking, we suggest that cancers with a robust causative linkage with bacteria (such as gastric cancer by *H. Pylori* and possibly CRC by *F. nucleatum*) develop as an aberrant phenocopy of bacteria-driven mucosal repair. As briefly outlined in the paragraphs below, a large part of the information reported in Section 3 (“F. nucleatum and CRC”) can be reorganized and interpreted within this conceptual framework.

### 5.1. Enhancement of Stem-like Features in Epithelial Cells

*F. nucleatum* elicits or amplifies, in CRC cells, stem-like traits, including motility/invasiveness and epithelial-to-mesenchymal transition, shared among malignancy, morphogenesis, and wound repair [146]. Central to this action is the activation of the Wnt/β−catenin cascade, possibly in conjunction with other niche-related signals such as Notch/RBPJ [75]. Activation of the Wnt pathway is a recurrent theme in bacterial carcinogenesis [16], and likens *F. nucleatum* to *H. Pylori* [21] and other pathogens linked to colorectal cancer, such as *Bacteroides Fragilis, and Salmonella Enterica* [15]. In these examples, β−catenin/TCF signaling is often initiated by molecular interactions (FadA-E cadherin, Fap2-Glc/GlcNac, CagA through GSK-3β [147], AvrA [148]) distinct from the canonical innate immune pathways. It is tempting to speculate that these interactions are part of a parallel and complementary microbial sensing system, specifically dedicated to “regenerative” epithelial signaling.

Besides increasing cancer cell “stemness,” *F. nucleatum* directly targets CR-CSCs via multiple interactions (CbpF/CEACAM1; Fap2-Gal/GalNac); *F. nucleatum* infection further increases the constitutively high Wnt activity of CSCs, while eliciting resistance to cell death and NF-kB- dependent chemokine release (see below 5.2) [68]. Likewise, *H. Pylori* directly activates Lgr5+ gastric stem and progenitor cells, leading to gland hyperplasia and remodeling [149], changes eventually conducive to malignant transformation.

### 5.2. Reparative Inflammatory Responses

Secretion of inflammatory mediators downstream of the master transcriptional regulator NF-kB has been frequently reported in CRC cells in response to *F. nucleatum nucleatum* [61] [65,68]. In a physiological repair setting, cytokines recruit leukocytes to the damaged site, while promoting epithelial regeneration [136,139,144]. In CRC, interleukin 4, a typical “type 2” cytokine involved in the resolution of inflammation and mucosal repair, acts on colorectal CSC as an autocrine factor that inhibits apoptosis and favors chemoresistance [150] and escape from T cell-mediated immunosurveillance [87]. Moreover, NF-kB synergizes with deregulated Wnt/β-catenin signaling in promoting stem cell expansion [116], dedifferentiation of mature colonocytes [128], and cancer cell survival [108] during colorectal tumorigenesis. *F. nucleatum* triggers NF-kB activation and the release of CXCL1 and CXCL-8 in CR-CSCs [68]. Likewise, *H. Pylori* activates NF-kB in gastric stem cells via the Wnt target Lgr4, and NF-kB transcriptional activity is simultaneously responsible for the proliferation of self-renewing stem cells and the upregulation of chemokine genes, which enables neutrophil recruitment [151]. Collectively, these observations underscore the intimate interlacement between inflammatory and growth/survival pathways operating in intestinal stem cell activation during both mucosal regeneration and cancer. This two-signal mechanism, which resembles the activation of naïve T lymphocytes (signal 1 = antigen receptor via tyrosine kinase signaling; signal 2: co-stimulatory molecules and inflammatory mediators via NF-kB) [152,153], guarantees that cell reactivity (ISC or T cell) is proportionate to the level of tissue damage or “danger” (Figure 3).

### 5.3. Downregulation of Adaptive Immunity

In coherence with the execution of a prototypical mucosal repair program, *F. nucleatum* inhibits adaptive immunity through the establishment of an immunosuppressive environment. This occurs via the direct engagement of immune checkpoint receptors TIGIT and CEACAM1 on T and NK cells [43,70], as well as through the NF-kB dependent release of neutrophil-recruiting chemokines (such as Il-8 and CXCL-1) from epithelial and stromal cells [65,68]. Neutrophils participate in tissue repair by releasing growth factors (such as vascular endothelial growth factor) and lipid mediators (such as lipoxins, resolvins, and protectins) that facilitate inflammation resolution and mucosal healing [154]. In this context, neutrophils also suppress adaptive immunity [155], and this is especially true within the tumor microenvironment [88]. Similarly, H. Pylori recruits neutrophils via stem cell-derived chemokines to establish chronic active gastritis [151].

## 6. Finale: Cancer from a Bug’s Perspective

*F. nucleatum* infection may promote colorectal carcinogenesis but is unlikely to initiate it. Instead, this oral pathogen preferentially lodges in CRC tissue and hitchhikes metastatic malignant cells (i.e., cancer stem cells) to their distant sites or reaches them through bacterial hematogenous dissemination. Far from being a passive “passenger”, *F. nucleatum* also contributes to nearly all the main hallmarks of CRC, favors malignant progression, and worsens clinical prognosis by impinging on the inflammation-stemness program that usually drives mucosal repair.

The conceptual framework whereby bacterial carcinogenesis recapitulates an aberrant repair process leaves unanswered the question of why *F. nucleatum*, instead of more aggressive enteric pathogens, is so strongly associated with CRC. Unlike *H. Pylori* in gastric carcinogenesis [156], *F. nucleatum* does not appear to set in motion the unresolved damage–regeneration cycle that eventually leads to malignancy [53,157]. From a different angle, a “regenerative” environment, either normal or neoplastic, could be particularly hospitable to this pathobiont. *F. nucleatum* displays high affinity towards the disaccharide moiety Gal-GalNac (also known as T antigen), whose abundance, as for other products of prematurely stopped protein O-glycosylation, is increased by hypoxia [158]. Thus, the Fap2–T antigen interaction may guide *F. nucleatum,* an obligate anaerobe, to poorly oxygenated tissues. Of note, hypoxia also occurs during wound healing [159], and *F. nucleatum* may contribute to oxygen depletion and pro-angiogenetic responses in periodontal disease [160]. In addition, proliferating ISC/CSCs may be particularly permissive to intracellular bacterial persistence due to elevated glycolysis [72], active Wnt signaling [161], and a longer lifespan compared to mature enterocytes. Along the same line of speculation, CR-CSC may create a sanctuary protected from T cell attack [87]; more generally, the immunosuppressive microenvironment characteristic of tumor stroma or a healing mucosa may favor bacterial colonization. Finally, a healing mucosa provides a weaker barrier to bacterial penetration [132], and the invasion of motile mesenchymal-like cells may favor bacterial dissemination. In short, *F. nucleatum* may have a propensity for the environment (wound/cancer) it eventually tends to reproduce. (Box 1). A similar fate has been proposed for *Helicobacter P*: this bacterium lodges in the junctional mucosa between the gastric antrum and corpus, where pH conditions are less extreme, and (in some susceptible subjects) tends to extend its “niche” towards the corpus (“antralization”) by inducing glandular damage, atrophy, and, eventually, substitution with metaplastic structures devoid of acid-producing principal cells [162]. Remodeling gastric mucosa is prone to malignant transformation, and, under the enduring genotoxic and proinflammatory action [163] of the pathogen, eventually progresses to gastric cancer.

Interestingly, advanced cancer tissue may become unhospitable to HP, thus justifying its disappearance [24,164] or substitution by other “passenger” bacteria, including *F. nucleatum* [165,166]. Conversely, *F. nucleatum* appears to be exceptionally well adapted to the cancerous environment, as confirmed by its emerging association with other non-CRC malignancies, such as pancreatic and breast cancer [27,167,168,169].

Box 1 ***H. Pylori* versus *F. nucleatum*.**

   Since the official recognition of *H. Pylori* as a causative agent for gastric cancer in 2012, probably no other bacteria have received more consideration than *F. nucleatum* as potential etiologic factors in cancer. The two pathogens, share differences and commonalities in their linkage to malignancy. Clinical and experimental evidence indicates that HP initiates gastric carcinogenesis via a chronic inflammation–atrophy–intestinal metaplasia–dysplasia sequence (the “Correa cascade”) in which the cytotoxin-associated gene A (CagA) plays an essential role. Cag-A expression is not always maintained in the malignant tissue and is not necessary for sustaining a neoplastic phenotype in established gastric cancer cells, suggesting a “hit-and-run” mechanism of carcinogenesis [24]. Accordingly, *HP* eradication effectively reduces gastric cancer risk, but less so in individuals harboring premalignant lesions before treatment [155]. *HP* is not necessarily less abundant in the normal adjacent mucosa compared to tumor tissue [27], in which it can be outcompeted by other microbial species. Overall, *HP* fits the " driver " role in the “driver–passenger” paradigm of bacterial carcinogenesis.

   Conversely, in spite of some genotoxic potential [51,57], *F. nucleatum* failed to initiate colorectal carcinogenesis in non-genetically predisposed mice, and data do not support the capacity of this pathogen to trigger a mucosal damage–inflammation–cancer cycle. Instead, *F. nucleatum* appears to be enriched in malignant tissues especially in advanced stages, and mechanistic studies highlighted a strong tumor-promoting capacity through multiple molecular interactions and signaling cascades, with no unique virulence factor. Consequently, it is unlikely that *F. nucleatum*-directed interventions will prove effective in preventing CRC, but they may help hamper its progression and metastasis [67,170]. (Box 2).

   In spite of these differences, commonalities have also emerged. At a molecular level, the similarity between the CagA-SHP2 and CEACAM1-SHP2 axes appears intriguing and worth further characterization. More generally, although operating in different phases of carcinogenesis, both bacteria trigger a similar array of signaling pathways (including Wnt/β catenin, and the NF-kB-Chemokine axis) and cellular programs encompassing epithelial stemness, survival, and secretory activity, all of which are overall related to wound healing and mucosal repair. Curiously, by promoting these aberrant tissue repair responses (i.e., respectively, mucosal “antralization” [161], and cancer progression), both *HP* and *F. nucleatum* appear to reproduce and expand the “niche” they have initially colonized.

Box 2 ***F. nucleatum* eradication and CRC progression.**

   So far, evidence that reducing *F. nucleatum* burden (i.e., by antibiotics or Aspirin) impacts CRC progression or response to therapy is limited to preclinical studies [67,170]. However, ongoing clinical trials addressing the effect of oral Metronidazol on postoperative chemotherapy (ClinicalTrials.gov Identifier: NCT04264676) or as a preoperative neoadjuvant agent (ClinicalTrials.gov Identifier: NCT05748145) on CRC outcomes may provide at least preliminary answers in the near future. On the other hand, antibiotics may actually increase the risk of CRC by altering the gut microbiota, and a positive association between high antibiotic use and cancer risk, especially in the proximal colon [171], where *F. nucleatum* positivity is most frequent, has been reported. The impact of the general antibiotic assumption on *F. nucleatum* abundance in the general population and CRC patients remains to be established.

## 7. Conclusions and Future Perspectives

We have here reviewed some of the most recent and relevant knowledge on bacterial carcinogenesis, focusing on the role of *F. nucleatum* in CRC. In doing so, we have revisited the old concept of cancer as a non-healing wound to incorporate the role of bacteria as drivers (*H. Pylori*) or passengers/amplifiers (*F. nucleatum*) of the aberrant mucosal repair program that leads to malignancy. This perspective centers on intestinal stem cells/cancer stem cells at the forefront of microbe–host communication and regulating normal and malignant mucosal growth. Importantly, as the interest in microbiota in cancer extends from CRC to other malignancies, the paradigm proposed here may receive further support and gain broader significance.

Despite the remarkable amount of information gleaned in mammals and lower organisms, much remains unclear about the signals and downstream pathways whereby bacteria and intestinal stem cells communicate with each other; likewise, the genetic and environmental factors that misdirect an evolutionarily conserved healing process towards cancer require further investigation. Most importantly, our knowledge of the potentially dangerous and protective bacterial species, their interactions, and the bacterial products and functions relevant to these activities is still in its infancy. This is a fascinating field of investigation, ripe for potential breakthroughs in the way we understand, prevent, and treat CRC and other cancers.
